# Dry growing seasons predicted Central American migration to the US from 2012 to 2018

**DOI:** 10.1038/s41598-023-43668-9

**Published:** 2023-10-26

**Authors:** Andrew Linke, Stephanie Leutert, Joshua Busby, Maria Duque, Matthew Shawcroft, Simon Brewer

**Affiliations:** 1 Department of Geography, University of Utah, Salt Lake City, USA; 2 LBJ School of Public Affairs, University of Texas at Austin, Austin, USA; 3 Robert S. Strauss Center for International Security and Law, University of Texas at Austin, Austin, USA; 4 University of Texas at Austin, Austin, USA

**Keywords:** Environmental social sciences, Climate-change impacts

## Abstract

Controlling for factors such as criminal violence and poverty, we tested if drier than usual growing season weather was a predictor of emigration from El Salvador, Guatemala, and Honduras to the US between 2012 and 2018. We focus on growing season weather because agriculture is a primary transmission pathway from the effects of climate change upon migration. We secured the migration apprehensions data for our analysis through a FOIA request to US Customs and Border Protection. Border Patrol intake interviews recorded the original home location of families that arrived at the southern US border. We used this geographic information to measure recent weather patterns and social circumstances in the area that each family departed. We found 70.7% more emigration to the US when local growing seasons in Central America were recently drier than the historical average since 1901.

## Introduction

Between 2012 and 2018, the US Border Patrol apprehended more than 1 million people traveling from El Salvador, Guatemala, and Honduras (hereafter the Northern Triangle of Central America, or “NTCA”)^1^. The arrivals were not exclusively young adults seeking employment opportunities. According to CBP, 29% of these apprehensions were families (“individuals in a family unit” is the formal designation)^1^. These demographics demonstrate the broad scope of the issue, and suggest that the relocation is often permanent rather than temporary.

Some have attributed increasing NTCA emigration rates to violence, limited economic opportunities, corruption, political instability, and, more recently, the adverse effects of climate change. Climate change has altered weather patterns making rainfall and temperature trends unpredictable. Climate change also intersects with and possibly accentuates other weather phenomena such as El Niño Southern Oscillation (ENSO) cycles. ENSO cycles are known to reduce certain crop yields in some regions^2^. El Niño generally brings drier than average weather to the region during key growing months^3^, impacts that are likely to become more severe as global temperatures rise^4^.

Scholars have found migration adaptations to droughts and changing weather patterns globally^5–7^ and in Asian^8,9^, African^10,11^ and Latin American^12–20^ case studies. However, while some policy-oriented reports and insightful single-country studies^21^ have suggested a link between environmental stress and recent NTCA migration, we currently lack a comprehensive, empirical analysis of these human–environment interactions. A recent review of climate factors as drivers of migration claimed that evidence for precipitation is “inconclusive”^22^; our findings speak directly to this evaluation.

Because no longitudinal household survey data is available to model NTCA migration dynamics, we analyzed US Border Patrol apprehension data covering fiscal years 2012 through 2018. We acquired these data through a Freedom of Information Act (FOIA) request. These unique data record *family apprehensions*, which we argue is a conservative estimate of total migration (the majority of arrivals from 2012 to 2018 were single adults^1^). We mapped the original home location of the 323,579 NTCA residents in the FOIA dataset, which allowed us to measure the environmental and social conditions surrounding their departure. In particular, we are interested in local rainfall and evapotranspiration during the recent growing season, which we measure relative to historical data from 1901. We used these data to test if drier than usual weather predicts emigration from NTCA to the US.

## Results

Controlling for various factors, including criminal violence and poverty, we found that dry growing season weather predicted emigration from NTCA to the US. According to our preferred model estimate, a department with particularly arid growing season weather saw 1.7 times more people travel to the US (*e*^0.535^) than areas with typical weather. Put differently, a growing season where the Standardized Precipitation-Evapotranspiration Index 3-month average (SPEI03) was  − 1.0 standard deviation (SD) or drier than the historical average led to a 70.7% increase in migration. Our "Methods" section describes the Bayesian Integrated Nested Laplace Approximation (INLA, models 1–8) and spatial two-way fixed-effects ordinary least squares regression (TWFE OLS, models 9–12) estimators that we used to obtain our results.

**Figure 1:**
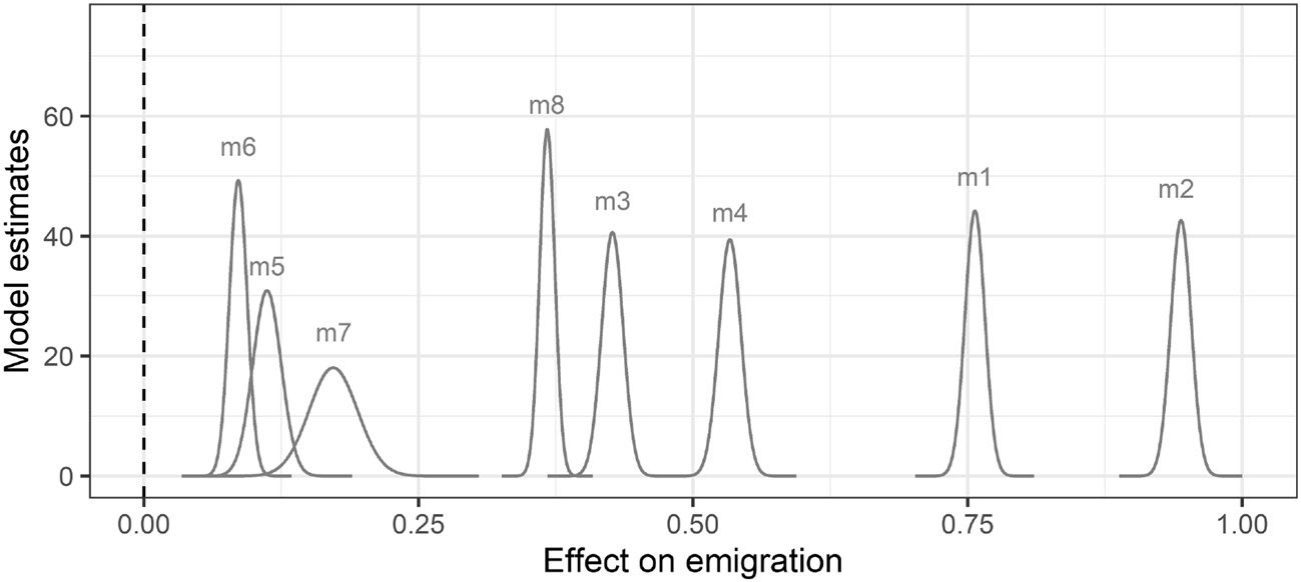
INLA estimates of dry growing season effects on emigration. The distribution of each curve contains all estimates of the effect from each model. The vertical dashed line indicates zero effect. Model 1 is a bivariate “null” model. Model 2 uses a different calculation of the expected emigration rate. Model 3 includes the homicide rate control variable. Model 4 uses all covariates. Model 5 uses a more conservative operationalization of dry conditions (SPEI03 ≤ −1.5 SD). Model 6 uses a longer time period for measuring dry conditions (SPEI12). Model 7 estimates the effect of dry conditions on the change in the emigration rate from the preceding year. Model 8 reproduces our main Model 4 results at the municipality level (*N* = 891).

A battery of robustness checks generally agreed with our main conclusion and are credibly distinct from zero effect (see Fig. 1). Supporting Information Tables S3–S10 present our full INLA model results with 95% credibility intervals. Model 1 uses only the dichotomous dry growing season variable, and we found 213.1% more emigration than in places with average weather (*e*^0.757^). Our models all incorporated an expected rate of emigration. Instead of department population size (used in other estimates), model 2 used the emigration rate in the preceding year to calculate an expected rate of travel to the US and the results were similar (*e*^0.940^ or + 257.0% emigration). Model 3 included the regional homicide rate as a covariate, which adjusts for a powerful alternative explanation for emigration. Our results were still credible and positive (*e*^0.427^ or + 53.2% emigration). In model 4, we added socioeconomic and vegetation health covariates (see "Methods"); this is our preferred main model, where we observed 70.7% more emigration when conditions were dry (*e*^0.535^). Table S3–S10 diagnostic statistics show that model 4 has the best fit (DIC = 27248.013). Interpreting the precision of the spatial random effect hyperparameter (Table S6: 1/2243.418 = 0.0004) shows that it explains the least variance in model 4, suggesting that dry weather and covariate fixed effects have considerable explanatory power. Among models that included covariates, preferred model 4 also had the lowest level of yearly temporal variance remaining (Table S6: 1/347.535 = 0.002).

An alternative (drier) threshold for SPEI03 as ≤ −1.5 SD gave us similar model 5 results (*e*^0.112^ or + 11.8% emigration). In model 6, we operationalized dry conditions using SPEI12 ≤ −1.0 SD instead of SPEI03 ≤ −1.0 SD. While the effect was smaller, it remained credibly distinct from zero (*e*^0.086^ or + 8.9% emigration). Model 7 used the change in the emigration rate from the preceding year as the outcome, and we still found that dry weather predicts emigration (*e*^0.173^ or + 18.8% increase in the emigration rate). This effect estimate is smaller than our preferred model 4. Instead of indicating the risk of emigration during droughts, it predicted *how much the emigration rate is rising* with drier weather conditions. Finally, recalculating the variables and running our estimates at the municipality level (see emigration rate maps at this scale in Fig. S1) gave us consistent results. As with the others, model 8 predicted greater emigration during dry growing season conditions (+ 44.3% emigration, or *e*^0.367^).

As we tested alternative operationalizations of the dry conditions dichotomous predictor, we also check our main results against a model using the continuous normal distribution of SPEI03. In a modified version of the main Fig. 1 INLA estimates we confirmed that incrementally wetter conditions – increasing SPEI03 values – reduce migration (see Fig. S9). A test for non-linear effects of SPEI03 across the range of values (low/dry to high/wet) also shows a spike in migration risk around  − 1.0 SDs and lower migration rates above 0.0 SDs (see Fig. S10).These results generally complement our finding that dry conditions increase migration.

Our within-unit TWFE OLS model results were generally similar (see Table 1). Controlling for all unobserved department, country, and year effects, dry growing seasons raised the average emigration rate 158.5 people per 100,000 people above the rate during average weather (model 9). Model 9 results are influential, considering the average emigration rate is 126.0 per 100,000 people (see descriptive statistics Table S1). If we used emigration rate change as the outcome, as in INLA model 7, the model 10 results show that a dry growing season leads to a 198.4 person per 100,000 increase in the emigration rate from the preceding year. These findings reinforce our main results and explain considerable variation in the relationship between environmental stress and migration from NTCA to the US (e.g., model 9 *R*^2^ is 0.63). We also estimated the spatial simultaneous autoregressive error models 11 and 12. The results are nearly identical to the within-unit effect of dry growing seasons on emigration (+ 158.05 and + 197.845 for emigration and emigration rate change, respectively).

**Table 1:** Alternative estimates of dry growing season effects on emigration (m9, m11) and emigration rate change (m10, m12) using TWFE OLS (m9, m10) and analogous spatial simultaneous autoregressive error models (m11, m12).

	m9: emigration rate	m10: Δ emigration rate	m11: emigration rate	m12: Δ emigration rate
(Intercept)	14.550	− 2.631	14.787	− 2.665
	(44.569)	(54.841)	(46.807)	(56.319)
Dry growing season	158.586**	198.400**	158.052**	197.845**
	(56.719)	(66.082)	(54.931)	(63.671)
λ			− 2.090***	− 2.028***
			(0.295)	(0.288)
Department FE?	Yes	Yes	Yes	Yes
Year FE?	Yes	Yes	Yes	Yes
Country FE?	Yes	Yes	Yes	Yes
Moran's I	− 1.163	− 0.905	− 2.385	− 2.385
Moran's I *p* value	0.878	0.817	0.991	0.991
R^2^	0.630	0.318	0.677	0.422
Adj. R^2^	0.560	0.166		
AIC			4676.339	4102.144
BIC			4924.237	4336.550
Deviance			3467425.049	3741617.385
Log likelihood			− 2275.169	− 1989.072
N	378	324	378	324

TWFE OLS; 54 observations are lost in model 10 and 12 due to constructing a rate change variable; standard errors in parentheses. ***p < 0.001; **p < 0.01; *p < 0.05; λ is the model spatial error parameter.

All of our models used three essential variables. We describe additional model covariates and their operationalization in "Methods". First, we secured our El Salvador, Guatemala, and Honduras emigration data using a FOIA request to CBP, an agency within the Department of Homeland Security. US Border Patrol detained 323,579 individuals traveling as part of nuclear families at the US-Mexico border between 2012 and 2018. These data measure people arriving through illegal border crossings between formal ports of entry. The number of arrivals is our outcome variable, but being a member of a household is a key selection mechanism in the data generating process. The arriving nuclear family could be some part of a larger household (e.g., a father and son with mother and another child at home). There are no formal empirical designations about asylum claims in our data, but most arriving Central American families would have claimed asylum upon apprehension.

In their interviews with the apprehended, officials asked where they were from. We used this geographic information to calculate the emigration rate per 100,000 people in each NTCA department (*N* = 54) per year. Then, we merge these emigration rates—our primary outcome of interest—with other administrative unit variables, including weather patterns. Our data measure families relocating is probably a conservative proxy for total migration. The impetus for uprooting an entire household for a perilous journal is almost certainly more powerful than for one member of a family to relocate and mail remittances. The Border Patrol reported that between fiscal years 2012 and 2018, nearly 30 percent of apprehensions at the US border were individuals in family units. More broadly, according to the International Organization for Migration, 43% of migration to Organization for Economic Cooperation and Development member countries during 2021 was broadly defined as “family migration” (including reunification); of that share only 17% were “accompanying family,” which is defined as “family members who are admitted together with the principal migrant”^23^.

**Figure 2:**
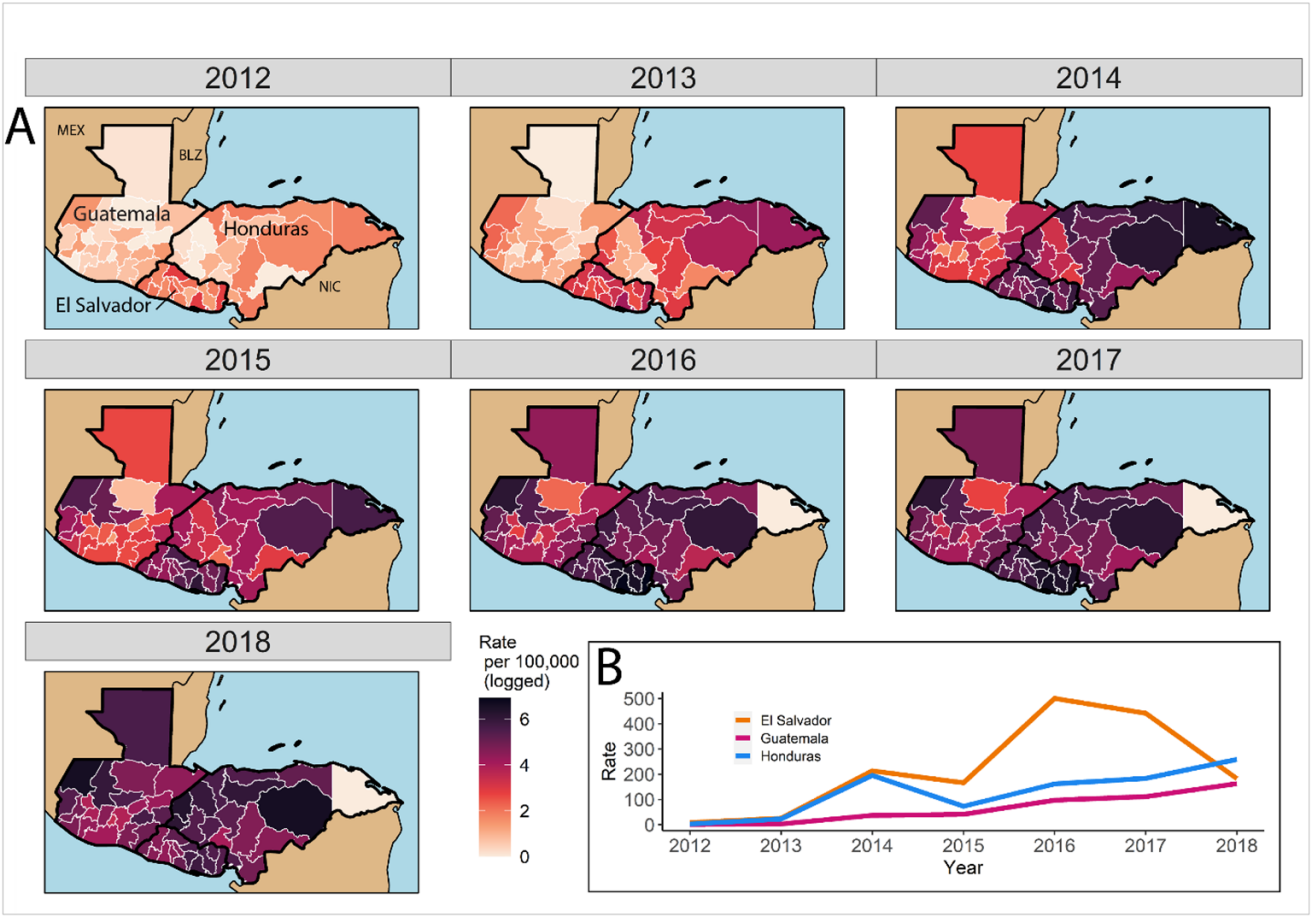
Emigration rates from El Salvador, Guatemala, and Honduras departments (*N* = 54) to the southern US border according to US Border Patrol intake interview data (**A**). Darker regions have higher emigration rates. Over time, emigration rates generally increase but illustrate some temporal and regional variation between 2012 and 2018 (**B**).

Figure 2A presents maps of NTCA annual emigration rates. There is considerable spatial heterogeneity, with some regions of western Honduras experiencing much higher emigration rates during 2013 than central and northern Guatemala (rates were more similar in later years, including 2018). Figure 2B shows that Emigration rates exhibited a consistent increase except in El Salvador, which experienced a rise through 2016, but subsequent decline afterward. Table S1 presents summary statistics for all variables and identifies the models that used each indicator. We also present municipality level (*N* = 891) emigration rates in Fig. S1 and confirmed our main model results at this scale. Additionally, we tested operationalizing the outcome variable as emigration rate change from the preceding year (see Fig. S2).

**Figure 3:**
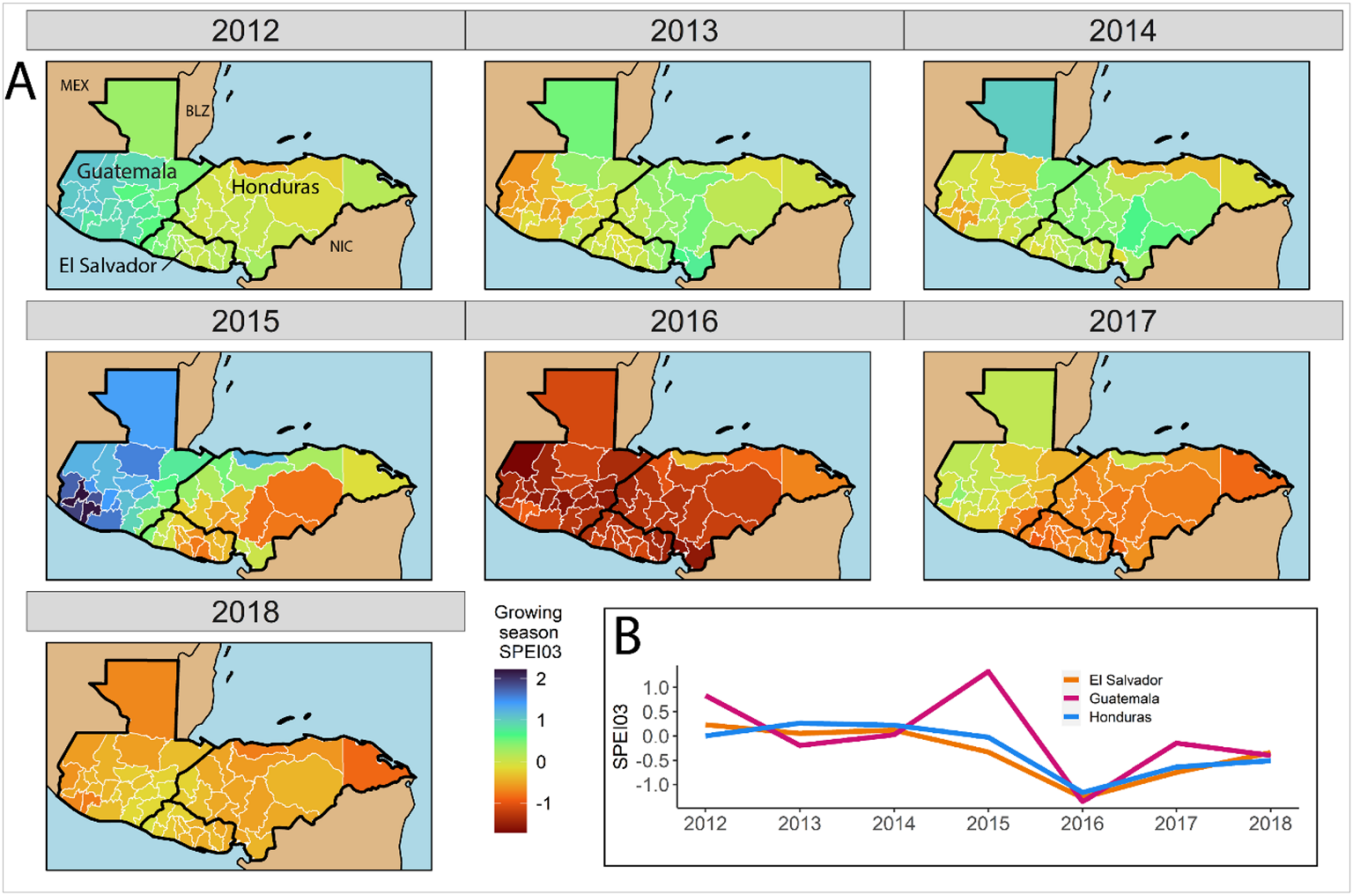
Department-level (*N* = 54) growing season Standardized Precipitation Evapotranspiration 3-month weather deviations from the historical average (**A**). Data source: Climate Research Unit TS 4.03. The SPEI03 data are operationalized as standard deviations. With some exceptions, SPEI03 values demonstrate a slight decrease overall from 2012 to 2018 (**B**).

Second, our weather data are from the Climate Research Unit (CRU) TS 4.03 time series from 1901 to 2018^24^. CRU data were calculated using historical empirical measurements and publicly-documented climatology and interpolations available from the data project^25^. We used a zonal statistic to calculate the mean 0.5-degree raster layer pixel SPEI value within administrative unit boundaries (either departments or municipalities). SPEI is expressed as SDs and accounts for temperature and hours of sunlight during the corresponding months, offering a more comprehensive measurement of weather conditions than rainfall alone^26^. We primarily relied upon a SPEI 3-month average (SPEI03), which compares a given period (e.g. January through March) to the regional long-term historical average for those months. Figure 3A shows the average growing season SPEI03 values across departments. In 2015, there was considerable spatial variation in growing season weather; southern Guatemala was much wetter than average, while eastern Honduras was much drier than usual. 2016 was significantly dry across all of NTCA, a trend reflected clearly in the SPEI03 time series (see Fig. 3B). We lagged the SPEI03 values so that the indicator captures environmental conditions before emigration. We coded drier than usual weather as a dichotomous variable if SPEI03 ≤ −1.0 SD, but also tested a threshold ≤ −1.5 SD.

The connection between arid conditions and failing harvests—the key transmission pathway between climate change impacts and migration—would likely manifest during the growing season. According to the Food and Agriculture Organization, the primary growing months in the region are May through November in El Salvador, May through October in Honduras, and April through December in Guatemala^27^. These ranges obscure regional variation. To achieve a highly precise local measurement of these critical growing season months sensitive to varying climatic zones, we used the Anomaly Hotspots of Agricultural Production (ASAP) phenology data^28^. ASAP identifies the growing season of the main crop in an area using a long-term average (2003–2016) of a ten-day MODIS Normalized Difference Vegetation Index (NDVI). These 1 km raster data identify an average calendar day (ranging from 1 to 365) when the growing season starts (NDVI grows above 25% of the ascending amplitude) and ends (NDVI drops below 35% of the descending trend). Fig. S3 presents the considerable regional variation in growing season start and end dates. As a robustness check, we tested using SPEI12 (see Fig. S4), which measures dry conditions for the entire year, instead of growing season SPEI03. The results generally agree.

Third, we used homicide rate data to control for the influence of violence on emigration (see Fig. S5). We obtained the Guatemalan records using a transparency request filed to the Interior Ministry (*Ministerio de Gobernación*). We gathered the Honduran homicide records from the Online Police Statistics System (*Sistema Estadístico Policial en Línea*). Finally, we coded the El Salvador data from the online homicide repository files published by the National Police (*Policia Nacional Civil*). While some homicides may go underreported, we are confident that these data serve as an effective proxy for exposure to violence. We calculated a murder rate per 100,000 people, merge these into administrative units, and lag the indicator by one year.

## Discussion

Existing research has shown that unusual weather variability and unpredictable seasons has contributed to migration globally^5–22^. We confirmed that this is also true for recent Central American migration to the US. In developing countries with economies heavily reliant on agriculture, declining harvest yields threaten farming livelihoods when investments surpass revenues^29–31^. These deficits force millions of households worldwide to adapt: “Negative shocks to agricultural productivity caused by climate fluctuations significantly increase emigration from developing countries”^32^.

The economy and the environment are tightly coupled across NTCA. In Guatemala, Honduras, and El Salvador, agriculture, including coffee, corn, and bean cultivation, employed 32%, 37%, and 30% of the working population in 2012, according to the International Labor Organization. These rates are proportionally higher in rural regions of each country. By 2019, the agricultural shares of employment nationally had fallen to 31% (Guatemala), 29% (Honduras), and 16% (El Salvador). Even people who are not farmers but who earn wages harvesting or processing agricultural goods could still lose their livelihoods as yields fall.

Closely comparing our data to NTCA farmers’ experiences reassures us that drier than average growing seasons and emigration were plausibly related. For example, in 2016, the Las Marias community in Olancho department, Honduras, showed journalists and relief workers dry wells that no longer supported their families’ crops^33^. In the same year, the Famine Early Warning Systems Network expressed grave concerns about hunger in Olancho and other nearby areas^34^. Our data reflect these conditions, with Olancho experiencing a drier than usual growing season (SPEI03 = −1.18 SD). Furthermore, Olancho’s 2016 emigration rate (379.8 people per 100,000) was above the 90th percentile (287.2 per 100,000).

Similarly, crops in San Miguel, El Salvador, withered without rain during 2016, and residents there reported to relief agencies that conditions were too dry even for alternative varietals^35^. San Miguel farmers eventually relied exclusively on emergency food supplies. As in Olancho, our data reflect these narratives. The average SPEI03 during the 2016 growing season was  − 1.41 SD that year, and the emigration rate was 624.1 people per 100,000 (three times the regional average). In another example, the Red Cross warned of dire drought conditions in Guatemala during the 2015 El Niño. During El Niño, Guatemala experiences *canícula* or *veranillo*, a phenomenon when it stops raining for several weeks or more during the rainy season (also called a “midsummer drought”)^36^. Among other departments, Huehuetenango is named in their reporting of “3.5 million people in need of assistance”^37^. Our data effectively captured the drought (SPEI03 = −1.7 SD), and we also observed a correspondingly high emigration rate (529.6 per 100,000 people). These illustrative examples demonstrate how lower than average rainfall impacts livelihoods and, in turn, plausibly raised emigration rates.

Many in NTCA supplement their diets with subsistence crops grown on small plots. Abnormally dry weather can lead to food security challenges through lost farm revenues among smallholders, diminished wages, or failing personal gardens. The World Food Program and International Organization for Migration recently called attention to rising food insecurity among nearly 3.5 million people living in the “dry corridor” that runs through all three countries^38^. In addition, ethnographic research and household survey data analysis confirm that food insecurity is a dire concern under drying weather conditions in the region^39^.

The adverse effects of climate change *beyond water shortages* also contribute to crop failures. Across NTCA, forecasts have shown reductions in the region's suitability for coffee, a crop that provides the largest share of rural employment^40^. For example, rising temperatures have contributed to plant diseases like “coffee rust” and aflatoxin^41^. Coffee rust is caused by a fungus (*Hemileia vastatrix*) that affects the coffee species most common in El Salvador, Guatemala, and Honduras (*Arabica*). Higher normally-cool and lower normally-hot monthly temperature averages reduce the latent period of the *Hemileia vastatrix* fungus. In 2013, severe coffee rust caused over $1 billion in damage^42^ and reduced yields by 16% compared with 2011–2012^43^. We analyzed potential evapotranspiration indicators (e.g., SPEI03) incorporating temperature trends that account for these ecological impacts.

Independent of agricultural trends in NTCA, global coffee prices fell after 2011 with a temporary spike in 2014–2015^44^ and only a recent increase in 2021. Coffee rust episodes and low average prices combine with short-term weather variability to undermine farmers' livelihoods. Rising fertilizer, equipment, and transportation costs, unfavorable international currency exchange rates, and a host of other production and supply-chain dynamics^13^ influence migration decisions in addition to the weather, but we accounted for such temporal trends in our INLA and TWFE OLS models.

Studies have found that environmental stress proxied by temperature and precipitation extremes is more likely to lead to *international* emigration from rural areas than *internal* migration to other domestic cities^45^. Such conclusions about the scope of migration suggest that planning to travel to the US from NTCA, rather than remaining in-country, is plausible for struggling farmers. Strong global community networks and anticipated climate-related wage changes may account for these differences.

Migration responses to climate change are complex. Under certain circumstances, relocating can be a highly productive adaptation strategy^31,46–48^. On the one hand, an intuitive scenario links drought to migration, which can occur through agriculture shocks: “municipal-level rainfall deficits relative to historical averages are an important predictor for both international and internal migration, especially in municipalities with predominantly rainfed agriculture”^19^. On the other hand, scholars have cautioned against reductive, deterministic, and overly-simplistic interpretations of this chain of events because migration responses to climate change are not inevitable. It is beyond the scope of this study, for example, to study so-called “trapped” households, who may lack the financial, institutional, or social capital that facilitates relocation^49–51^. Of course, securitized borders also make international migration dangerous and expensive^52^. Still, this fact only suggests that our family apprehensions data are a conservative estimate of how severely droughts have affected NTCA communities.

Beyond accounting for secular temporal trends in our analysis, our study accounted for influential national-level political circumstances that might correlate with migration. El Salvador, Guatemala, and Honduras have long histories of autocratic rule, for example, and the legacies of US interventions have contributed to chronic political instability and weak governance. The three countries currently have limited state capacity, and investments in the provision of public goods are low. In 2018, the NTCA countries ranked last in Latin America for collecting tax revenue on GDP, according to the World Bank, which undermines effective governance.

In addition to the impacts of climate change, criminal violence and insecurity were among the most convincing alternative explanations for emigration from Central America to the US. In 2018, El Salvador, Honduras, and Guatemala had murder rates of 51.0, 40.0, and 22.4 per 100,000 people, respectively, compared to 5.0 in the US^38^. According to a 2015 US Senate Homeland Security and Government Affairs report, violence and insecurity topped the list of causes for NTCA migration^53^. The Internal Displacement Monitoring Centre reported that 242,000 and 247,000 people in Guatemala and Honduras, respectively, were displaced by violence as of 2020 (statistics are not available for El Salvador). Population survey research with over 49,000 respondents across Latin America found that the probability of “seriously considering family migration to the US” was 30% higher among families who were violence victims than households who were not^54^. Yet, there is evidence in some Honduran research that out-migration is primarily a function of material conditions (access to service and human capital accumulation) and less a result of high crime rates^55^. In our analysis isolating the impact of dry weather, we use official national police agency homicide data to control for these effects of violence.

Tables S5–S10 show that the INLA model posterior estimate for homicide rate is always reliably positive and distinct from zero. The effect on emigration is relatively small, however. The relationship also cannot be interpreted as plausibly exogenous from social context (as is the case for SPEI03). For example, in preferred model 4 (Table S5), every additional homicide per 100,000 people increases emigration by less than 1.0% (*e*^0.005^). Operationalizing the homicide rate using a binary indicator for departments that lie above the average (instead of the population normalized rate) confirms this relatively low magnitude effect (see Table S11). Holding covariates at their mean, a department with more violence than average will have 20.5% (*e*^0.187^) more emigration to the US. In these models, the credible effect of dry weather remains (*e*^0.662^) and has a stronger influence on emigration rates than violence.

Our results are robust, yet data limitations present opportunities for further research on this important topic. We use FOIA Border Patrol data because comprehensive, cross-national, and longitudinal household survey data are not available in the NTCA countries. Such data would allow scholars to evaluate our findings at a more granular level. One could definitively establish, for example, whether farming households suffering financial losses during droughts were more likely to be the same people arriving at the US border. More generally, the data generating process in our analysis begins with US Border Patrol apprehensions. Selection into the database requires successfully completing a long and arduous journey. Future research about NTCA migration during droughts could focus on communities relocating domestically, though such data may be difficult to obtain.

## Methods

In this section, we outline our model estimation methods and describe additional socioeconomic and environmental covariate data. These additional covariates were less centrally important to our study than emigration rates, growing season weather deviations, and homicide rates.

### Model estimation

First, we use Bayesian Integrated Nested Laplace Approximation (INLA) models to estimate the effect of weather patterns on emigration. Specifically, we estimate the number of emigrants (${Y}_{ij}$) per department ($i$) and year (*j*) as count data, assuming that they are conditionally independent Poisson distributed: ${{Y}_{ij}\sim Pois(\theta }_{ij}{E}_{ij}$), where ${E}_{ij}$ is the expected number of emigrants from administrative unit *i* per year *j*. We calculate ${E}_{ij}$ using both population size and the baseline emigration rate in the department in 2012 (the results are similar). A region’s relative emigration rate ${\theta }_{ij}$ is: $log\left({\theta }_{ij}\right)={\beta }_{0 }+ {\beta }_{1}{X}_{1ij}\dots {\beta }_{p}{X}_{p_{ij}}+{\varepsilon }_{ij}$, with intercept ${\beta }_{0}$ and ${\beta }_{1\dots p}$ vector of effects for ${X}_{1\dots p_{ij}}$ covariates (including weather deviations and additional covariates). Error ${\varepsilon }_{ij}$ has three components (it is a separable space–time model): spatial effect, temporal effect, and unstructured noise in ${\varepsilon }_{ij}={u}_{i}+$ ${v}_{j}+{w}_{ij}$. Spatially structured effect (${u}_{i}$) is modeled as a conditional autoregressive Besag-York-Mollié (BYM) model^56^. Spatial error is conditioned on neighbors and normally distributed with a mean derived from the mean error of the set of neighbors and a variance given by ${\upsigma }_{u}^{2} : u_{i} |u_{ - i} \sim N\left( {\overline{\mu }_{{\delta_{i} }} ,\frac{{\sigma_{u}^{2} }}{{n_{i}}}} \right)$. Temporally structured effect (${v}_{j}$) we model as an autoregressive-1 (AR1) model to account for the yearly trends in Figs. 2, 3 (panels B). Temporal error is conditioned on the error of the previous time step adjusted by AR correlation coefficient ($\rho$): ${v}_{i} \sim \rho ({v}_{j-1},{\upsigma }_{v}^{2})$. All remaining error is modeled as normally distributed noise in ${w}_{ij} \sim N(0,{\upsigma }_{{w}}^{2})$. Model 1–8 INLA estimations reported in the “Results” are fit using the ‘R-INLA’ package in R^57^. The estimates presented in Fig. 1 show the posterior distributions of the dry growing season effect. Tables S3–S10 present the point estimate (mean) of the weather effects with 95% credibility intervals. 

Second, we use within-unit two-way fixed-effects ordinary least squares (TWFE OLS) regressions. Our strategy is to consider weather indicators as plausibly-exogenous treatment variables with an as-if-random assignment to observations. Using fixed effects for year and department, we constrain the probability of treatment assignment to the known likelihood of extreme weather events in any region. In a controlled experiment, the analogous probability of treatment assignment is also known (it is zero or one). The calculation of SPEI achieves this by comparing past weather in an area to that region’s observable historical weather trends. We view these models as a robustness check against the INLA results. Specifically, the TWFE OLS estimates: ${{Y}_{ij} \sim {\beta }_{0} +{\beta }_{1}{X}_{i}+ {\beta }_{2}C+{\beta }_{3}j+{\beta }_{4}i +\varepsilon}_{ij}$ with emigration rate *Y* in observation *i* and year *j* affected at ${\beta }_{1}$ by dichotomous dry growing season variable ${X}_{i}$. Coefficients ${\beta }_{2}$, ${\beta }_{3}$, and ${\beta }_{4}$ capture any unobserved confounding migration influences in country (*C*), year (*j*), and department (*i*) fixed effects, respectively. The remaining stochastic error is captured in ${\varepsilon }_{ij}$. We do not include additional covariates in this estimation because any department-year variable would be redundant and not alter the main ${\beta }_{1}$ effect. We use TWFE OLS estimates in "Results" Table 1 models 9–10. We use a global Moran’s I with first-order queen contiguity weights statistic to test if model residuals are clustered, which would violate basic OLS assumptions. Finally, Table 1 models 11–12 replicate the straightforward TWFE OLS but in a spatial simultaneous autoregressive error estimation using ‘spdep’ in R. Table 1 shows that the autoregressive error models have a better fit than the baseline TWFE OLS estimates. 

### Covariate data

Our analyses include several covariates that are less critical to our results than the weather, migration and violence data (see "Results"). Table S1 presents summary statistics for the following three variables. Table S2 presents the statistics for the municipality-level models that also use these variables. Diagnostic statistics reported in Table S6 show that including these covariates improves the fit of our model. The covariate data are proxies for key influences upon the relationships between weather, agriculture, and migration. Figure 1 model 1 results show that excluding these variables from our analysis only changes the magnitude of the effect estimate and does not change our general conclusion that dry conditions increase the likelihood of migration.

First, the relationship between weather variability and emigration is probably most substantial where farming is common. We therefore measure the annual average area of each administrative unit classified as cropland by ASAP^28^. This cropland control also has the benefit of adjusting our effect estimates to account for the major type of agriculture. Specifically, these data would reflect short-term shifts in vegetation (e.g., annually harvested food crops); pixels designated as actively harvested cropland would not be timber forests. These 1 km pixel values record the area designated as cropland, and we average those values within administrative territories. Finally, we lag these values by one year to match the SPEI03 operationalization (see Fig. S6).

We also use a Normalized Difference Vegetation Index (NDVI) value measuring vegetation health. Suppose other forces reduce vegetation health in an area, such as infrastructure development or irrigation changes. In that case, our estimates of dry condition effects would be most robust with an NDVI control. NDVI data are from the National Oceanic and Atmospheric Administration Advanced Very High Resolution Radiometer (AVHRR) at 1.1 km resolution^58^. NDVI values range from  − 1.0 to 1.0, with higher values indicating healthy plants. Raw LTDR v5-AVHRR values are scaled from  − 1000 to 10,000 for radiometric and atmospheric corrections. We lag NDVI by one year (see Fig. S7).

Third, the general poverty level in an area could be an alternative explanation for emigration. As a surrogate measurement of socioeconomic status (SES), we use night-time light emission (NTL) remote sensing data. Existing research^59^ establishes a general precedent for this. In Latin America, Colombia research finds that NTL are positively correlated with socioeconomic status in both rural and urban areas^60^. We settled on these data because lighting has a cost that relatively wealthier households will be able to afford. Specifically, we use annual Visible Infrared Imaging Radiometer Suite (VIIRS) cloud-free coverage data^61^ measures 500 m pixel-level luminosity that we aggregate within administrative units using a mean zonal statistic. Fig. S8 maps the VIIRS SES proxy across regions.

### Supplementary Information

Supplementary Information.

## Data Availability

The datasets generated and/or analysed during the current study are available in the Harvard dataverse repository: https://dataverse.harvard.edu/file.xhtml?fileId=6414736&version=DRAFT.
