# Supplementary material for: Dry growing seasons predicted Central American migration to the US from 2012 to 2018
Source: Sci Rep. 2023 Oct 26;13:18400. doi: 10.1038/s41598-023-43668-9 (PMC10603058; doi:10.1038/s41598-023-43668-9)
Supplement: Supplementary file 1 — Supplementary Information. [file 41598_2023_43668_MOESM1_ESM.docx]

**“Dry Growing Seasons Predicted Central American Migration to the US from 2012 to 2018”**

Supplementary material

**Fig. S1.** presents the distribution of emigration rates across municipalities (*N* = 891) rather than departments (*N* = 54). See **Results** in the main text for an explanation of this robustness check. We operationalize growing season SPEI03 and other covariates at this level for the results presented in main text **Fig. 1.** (model 8). There are missing data at this fine spatial resolution (mapped in gray). As expected, these **Fig. S1. (A)** patterns generally match the distribution of emigration rates at a relatively coarse department-level spatial resolution. Over time (as in **Fig. 2. (B)**), the emigration rate rises in **Fig. S1. (B)**.

**Fig. S2.** presents an alternative operationalization of the emigration rates (the primary outcome of interest). Rather than modeling growing season SPEI03 effects for emigration in a given year, we also estimated dry conditions' effect on the emigration rate *change from the preceding year*. Without data for 2011, there are missing values for 2012. Results using this operationalization appear in main text **Fig. 1.** model 7. Among other years, we see unique subnational patterns in 2016, when the emigration rate rose considerably in parts of El Salvador and fell or remained nearly constant in other areas (e.g., northern Guatemala).

In **Fig. S3.** we present the average growing season start **(A)** and end **(B)** dates in each department according to the ASAP phenology data. Some department growing seasons begin near the end of one year and end during the next. In general, there is considerable subnational variation in the timing and duration of the growing season. Main text **Fig. 1.** results for model 6 use a year-long SPEI12 dry conditions operationalization of weather variability (**Fig. S4. (A)** maps these distributions) that is not restricted to the temporal window shown in **Fig. S3.**

**Fig. S4.** SPEI12 data corresponds with main text **Fig. 1.** model 6 results. These findings check whether the designation of growing seasons months using ASAP phenology data (see **Fig. S3.**) could influence our findings. Considerable spatial – **Fig. S4.** (**A)** – and temporal – **Fig. S4.** (**B)** – variations across NTCA are visible. For instance, in 2018, there were approximately -1.0 SD dry conditions in western Guatemala and southern Honduras but wetter than usual conditions (roughly +1.0 SD) in northern Honduras. Comparing years, it is clear that 2015 was relatively wet (especially in Guatemala) while during 2016, nearly all areas experienced much drier conditions than usual.

The most substantial alternative explanation of emigration across NTCA is criminal violence. **Fig. S5. (A)** presents a map of homicide rates across the region by department and year, according to official national reporting agencies in each country. Central Guatemala consistently has relatively low homicide rates compared with other areas. In contrast, El Salvador and North Central Honduras have persistently high murder rates over time (**Fig. S5. (B)**). The models incorporating these covariates are main text **Fig. 1.** models 3-8.

Changes in vegetation health could result from human activity in addition to drier than usual weather. These changes could influence emigration rates independent from the effects of unpredictable weather (e.g., infrastructure development encroaching on farmland is an alternative reason a person might leave whether or not it has been dry). In **Fig. 1.** model 4 estimations, we include a covariate measuring the change in average NDVI values across departments (see the **Covariate data** section of the **Methods** section**)**. **Fig. S7. (A)** presents the distribution of the NDVI change variable across departments (see **Fig. S7. (B)** for change over time). These data are used in **Fig. 1.** model estimates 4-8.

A link between unpredictable weather patterns and emigration is most plausible in areas where farming is the most common livelihood. We take this into account with the ASAP cropland raster layer and measure the average percentage of each actively cultivated department. Unsurprisingly, key cities like Guatemala City have very low shares of farmland compared to places like coastal El Salvador or northern Honduras (see **Fig. S6.**). This control is included in main text **Fig. 1.** model 4-8 results.

Poverty is an additional condition that drives people to emigrate and seek new livelihoods. As a proxy for regional SES disparities, we use nighttime light emissions data gathered by satellite instruments. Each raster layer pixel has a luminosity variable and we use a zonal statistic to measure the department average pixel value. The distribution of this SES proxy is mapped by department and year in **Fig. S8. (A)**. While drastic changes are not apparent over time in **Fig. S8. (B)**, subtle differences over large periods are evident. As expected, major metropolitan areas are comparatively well-lit and generally relatively wealthy compared to rural areas. Guatemala City and San Salvador are identified, for example. (Tegucigalpa is less prominent because it is in a relatively large department that contains peripheral rural towns distant from the capital.) As shown in **Tables S6-S10**, we use this covariate in models 4-8.

To demonstrate the robustness of our main findings we also re-ran a version of the INLA model that produced main text **Fig. 4.** estimates using the continuous normal distribution of raw SPEI03 values. **Fig. S9.** model m9 results (following models m1-8 in the main text) capture the effects of a 1 SD increase in SPEI03. As conditions are wetter compared to the historical average, migration decreases. The -0.25 estimate is reliably distinct from zero. This finding complements our core finding; instead of dry conditions increasing migration, wet conditions reduce departures. Further confirming this result, a spline (see **Fig.S10.**) illustrates potential nonlinear effects of SPEI03 on migration. The impact of dry SPEI03 values near -1.0 SD is clearly positive and distinct from zero effect. As expected, positive SPEI03 values capturing wetter conditions have a negative effect.

**Table S1** presents descriptive statistics summarizing all of the variables used in our analysis. The final column identifies the models that use each variable (these correspond with main text **Fig. 1.**). All variables illustrate intuitive distributions. For example, calculating the alternative change in emigration rate outcome variable for model 7 results in some negative values where fewer people left than the preceding year. The NDVI (AVHRR) change variable shows that some regions saw reduced vegetation health, but others experienced improvements. Even the nighttime lights (VIIRS) SES proxy captures some remote (perhaps protected) rural areas with little infrastructure development.

**Table S2** contains the summary statistics for the municipality-level analysis (main text **Fig. 1.** results model 8). As expected, the number of people leaving municipalities is, on average, much lower than among departments. However, other variables vary slightly as well. For example, it is realistic that the maximum homicide rate (3,073 per 100,000 people) in one particularly violent city could be far higher than a maximum department-level homicide rate (196 per 100,000 people) where a regional average includes more peaceful areas.

In **Table S3-S10**, we present the full model results from the estimations producing main text **Fig.1.** in the **Results** section. Each table caption contains references to the main model numbers 1-8. The effect upon emigration is reliable when a variable has a 95% credibility interval that does not include zero. Deviance Information Criterion (DIC) values summarize model fit. **Table S6** shows the lowest value among the results and is therefore the preferred model 4 effect reported in the main text. **Table S7** presents model 7 results with a lower DIC value, but this is the outcome's alternative emigration rate change coding and cannot be compared directly.

Spatial (administrative units) and temporal (year) random effect model hyperparameters are included in our INLA models. One is independent and identically distributed (IID) normal, the second is structured (see **Model estimation** in **Methods** with $u_{i}$ for regions and $v_{j}$ for space and time, respectively). The precision of hyperparameters are interpreted as 1/variance. To use the example of preferred main text model 4 (see **Table S6**), we calculate 1/2243.418 = 0.0004, which is the lowest level of spatial variance across models. The temporal effects control for yearly serial correlation in the time-series component of the dataset. Interpretation of precision for the year random effect hyperparameter is similar. Using **Table S6** values again, 1/347.535 = 0.002. Unsurprisingly, in the fine-resolution municipality level models, variance is the highest (**Table S10**: 1/0.031 = 32.25). **Table S11** presents a different operationalization of the homicide rate covariate using a dichotomous coding of whether departments were above or below the average across all three countries.

**
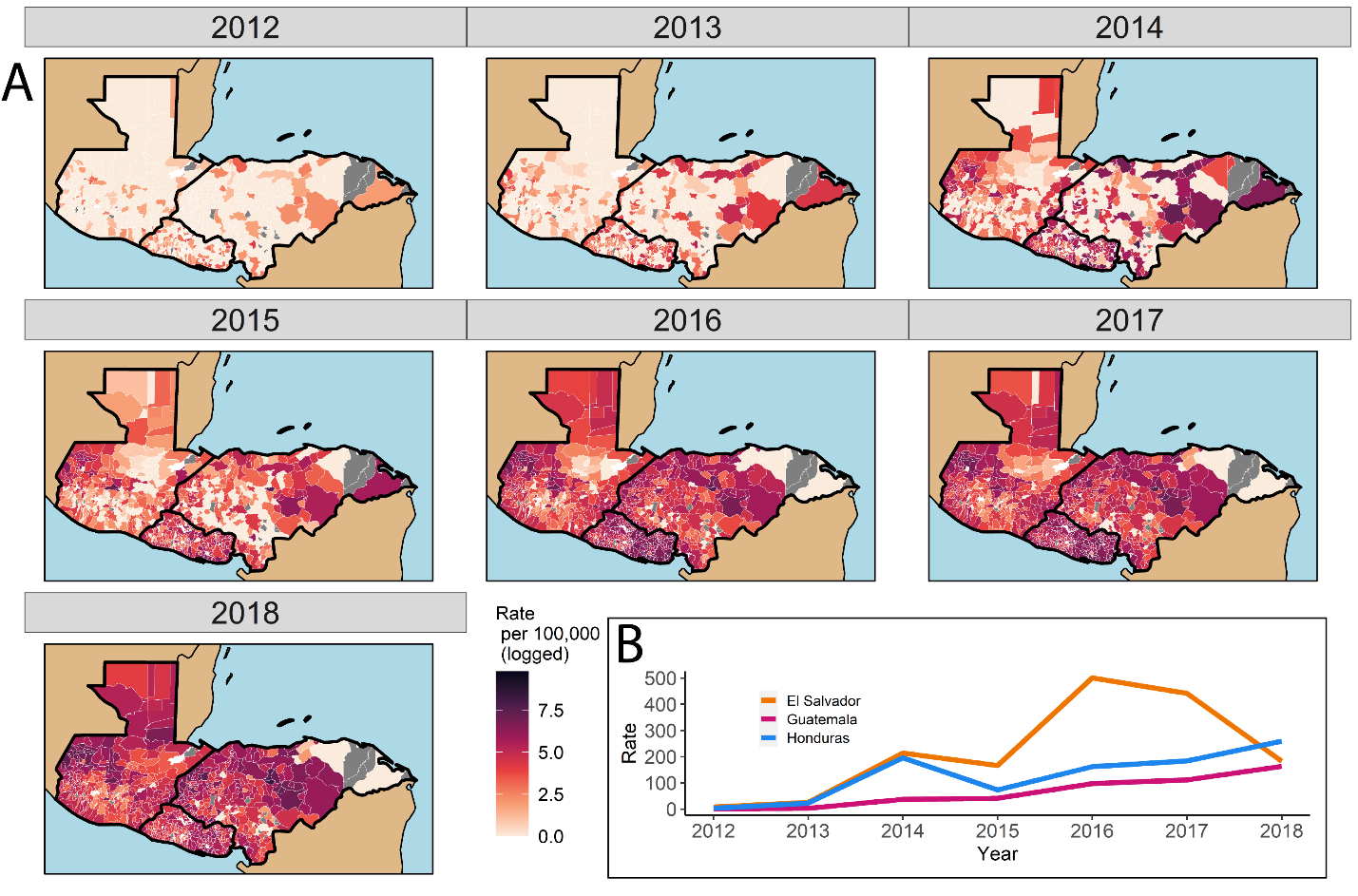
**

**Fig. S1.** Municipality level (*N*=891) emigration rates **(A)**. See corresponding main text **Fig. 2. (A)** for the department level map and also **Fig. 1.** model 8 results. We model total emigration but map the rates for presentation here. We account for emigration rate changes over time **(B)** in our modeling.


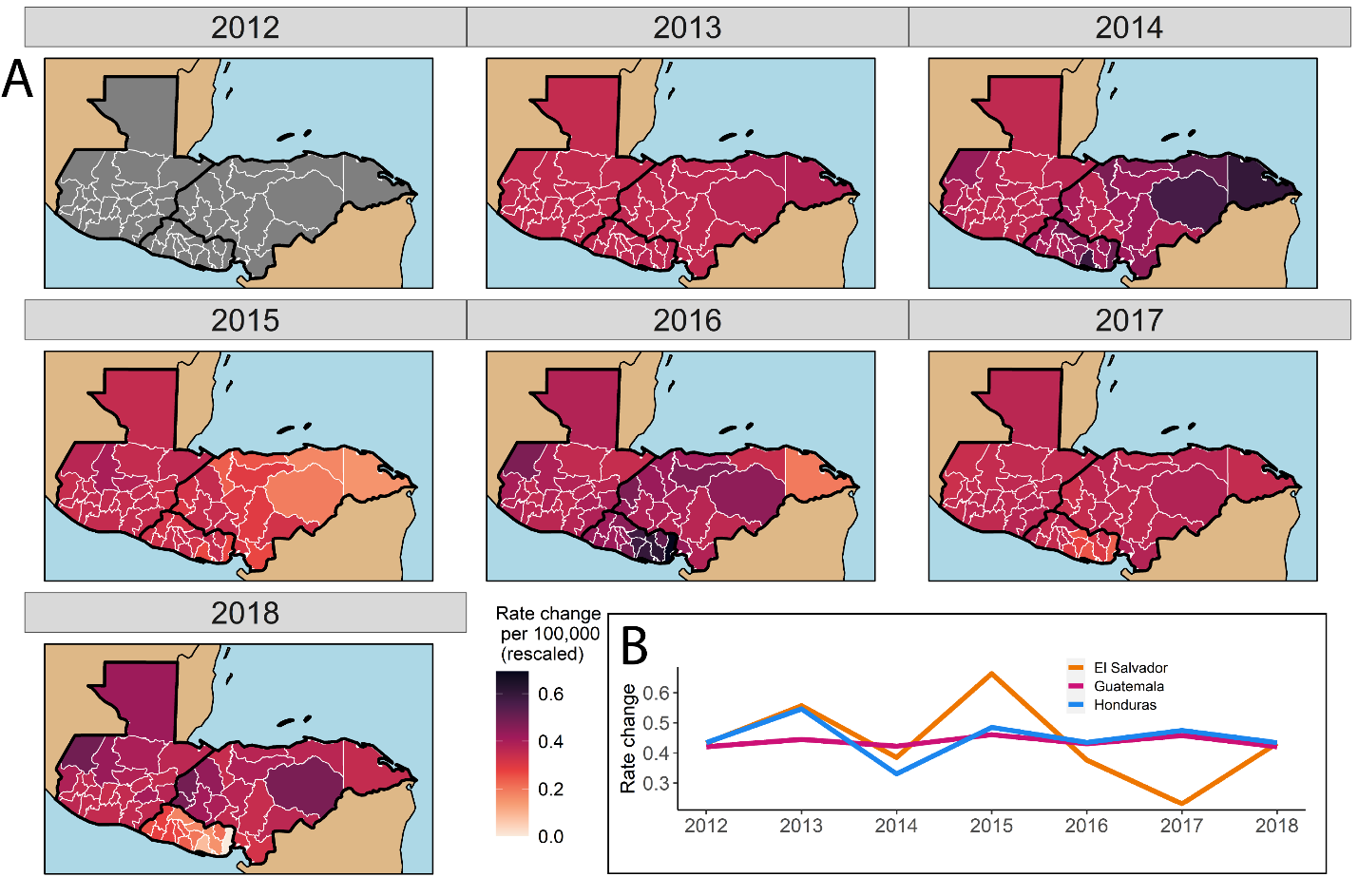


**Fig. S2.** By department and year, the change in emigration rate from the preceding year **(A)**. See corresponding main text Figure 1 model 7 results. Because we need a baseline to measure change, 2012 values are missing. We take clear temporal trends **(B)** into account in our models.

**
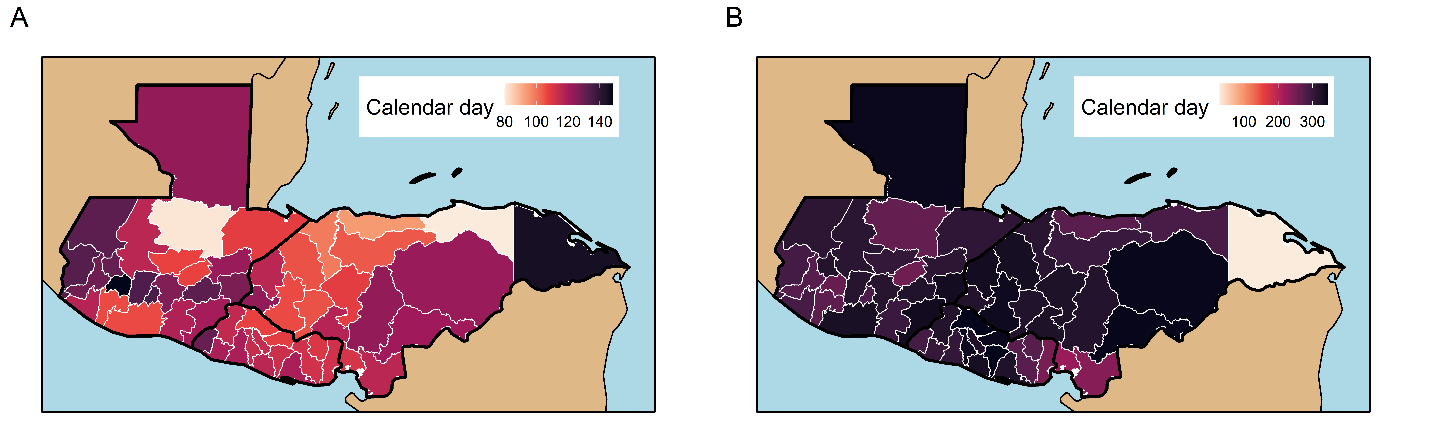
**

**Fig. S3.** Growing season start **(A)** and end **(B)** dates according to ASAP phenology calculations using 2003-2016 MODIS NDVI data. We use these dates to operationalize all SPEI03 variables so that they measure weather conditions during the growing season.

**
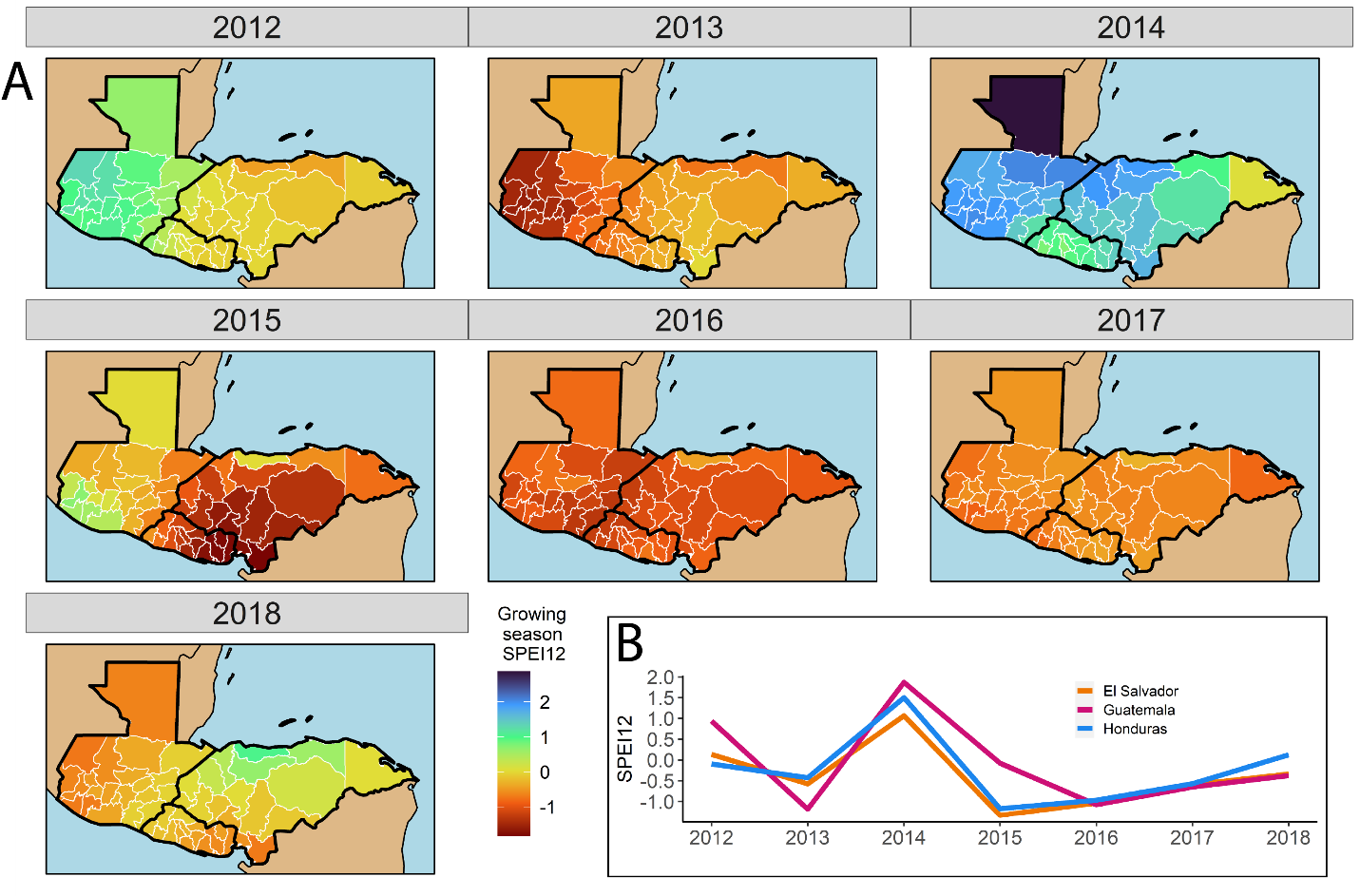
**

**Fig. S4.** By department and year **(A)**, an alternative operationalization of dry conditions (SPEI12 instead of SPEI03) without focusing exclusively on growing season months. For comparison, see main text Figure 3. These data are used in main text **Fig.1.** model 6 results. 2013, 2015, and 2016 were particularly dry across most of the region **(B)**.

**
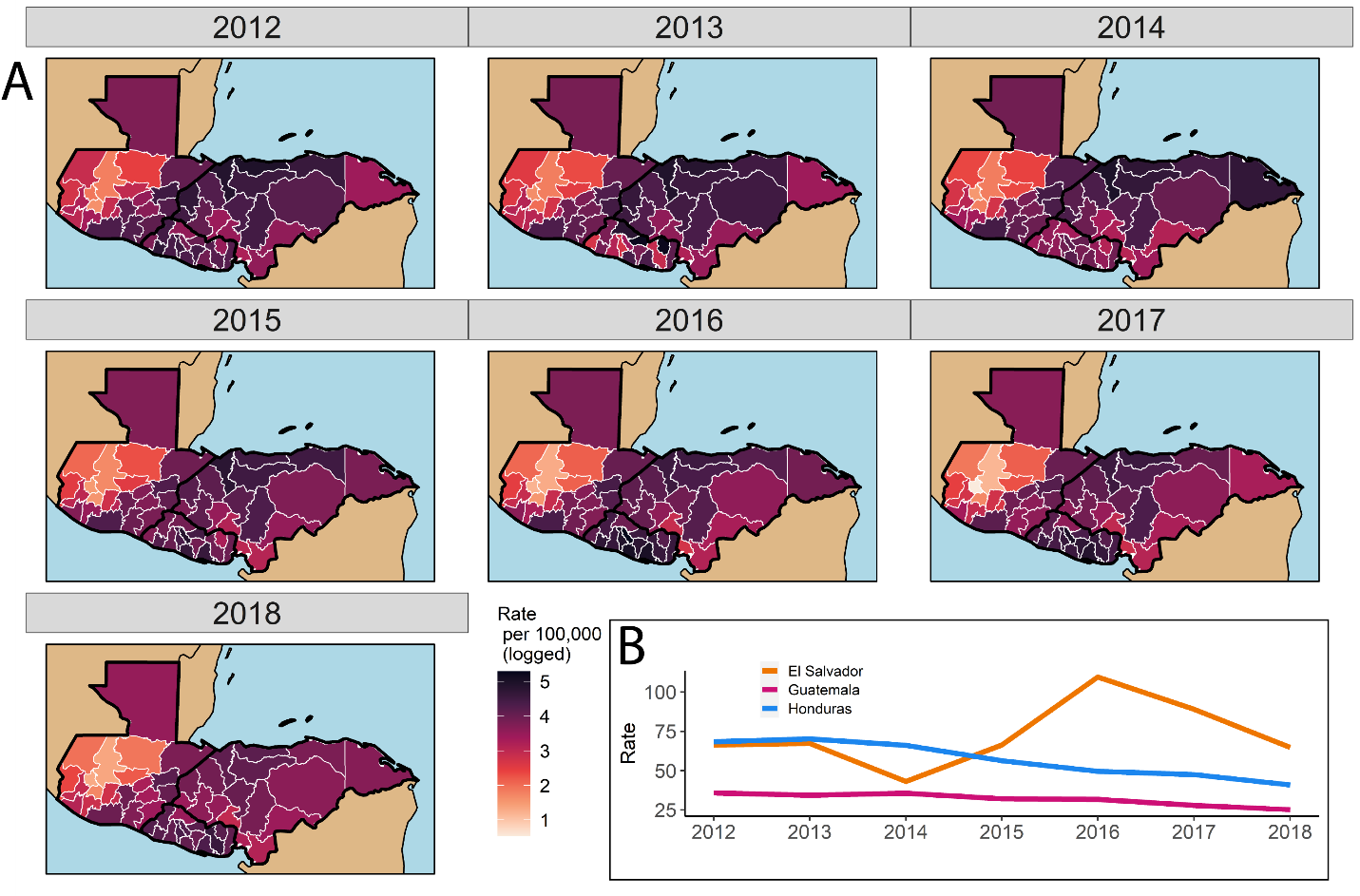
**

**Fig. S5.** By department and year **(A)**, homicide rates in NTCA according to official national reporting agencies. This alternative explanation for emigration is included as a control in main text **Fig. 1.** model 4-8 results. We control for considerable baseline homicide rate variation among countries and over time **(B)** as an alternative driver of migration.

**
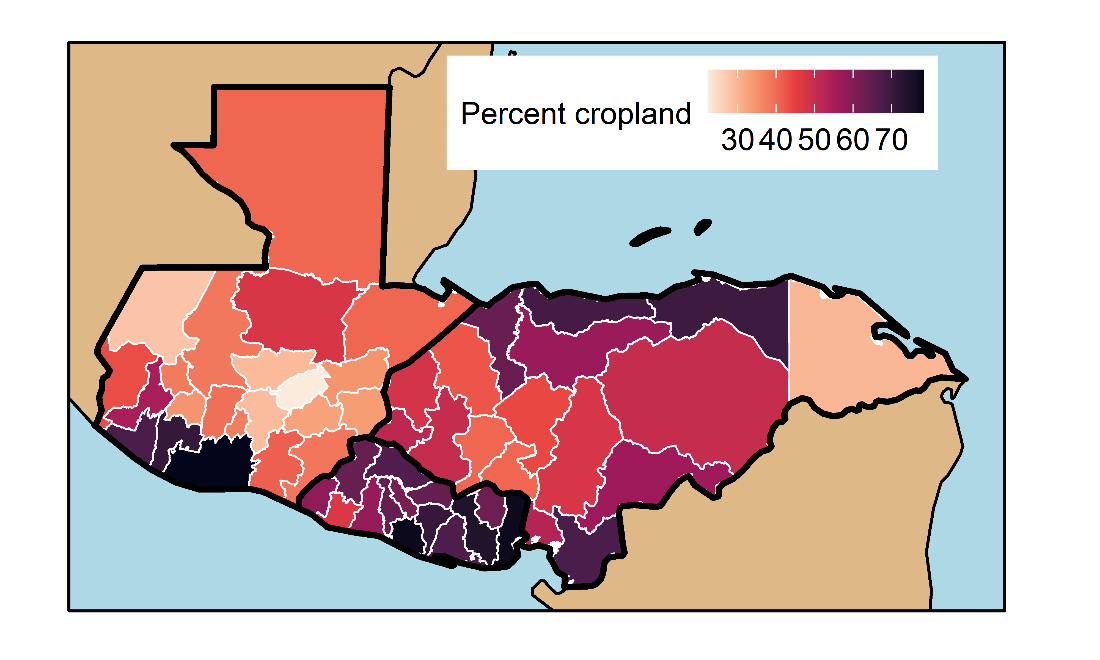
**

**Fig. S6.** Percent cropland in departments across the study area according to ASAP. We use these rates of agricultural productivity as a covariate in main text **Fig.1.** model 4-8 results.


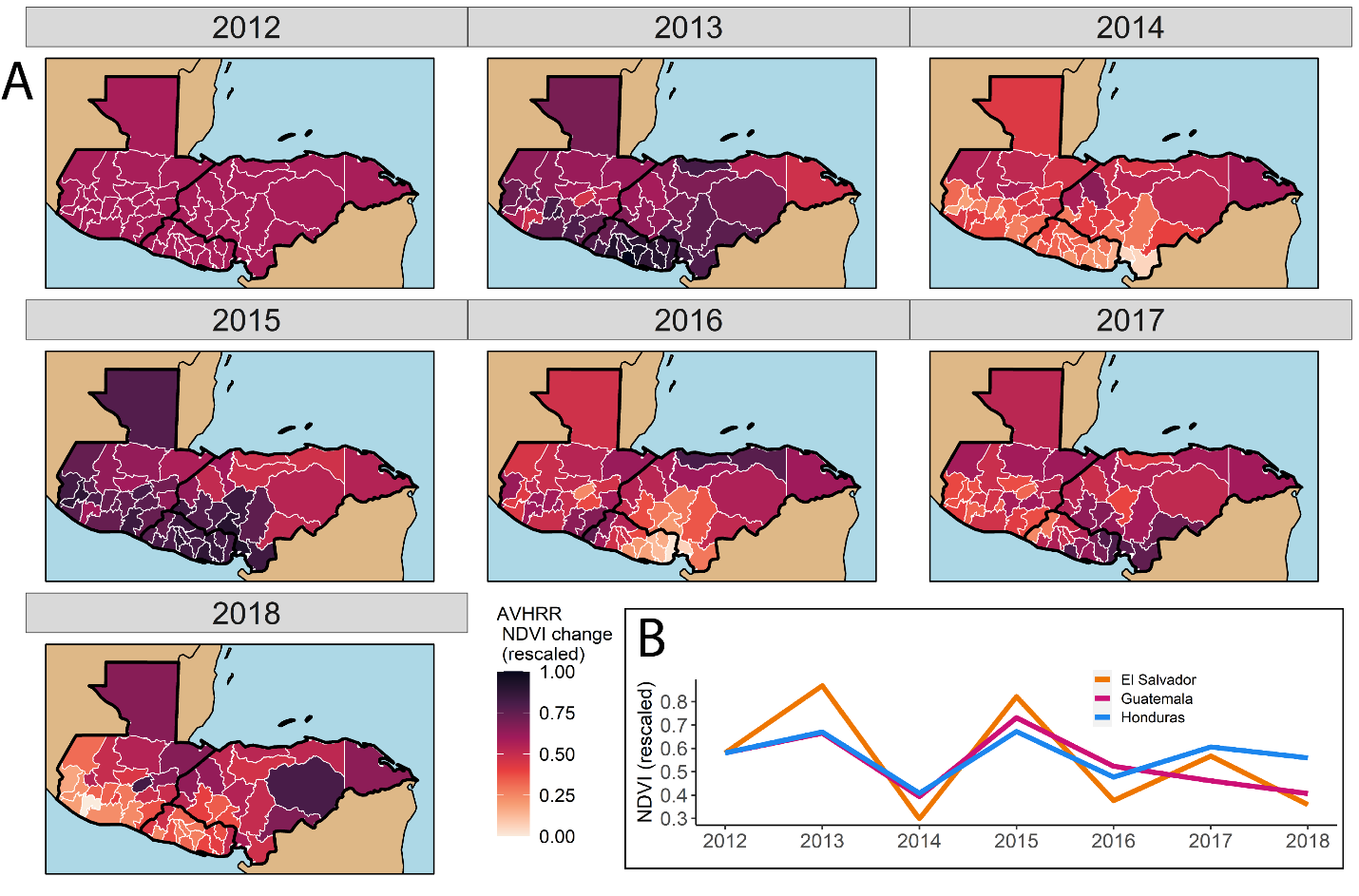


**Fig. S7.** By department and year **(A)**, annual change in AVHRR NDVI (rescaled 0-1 for estimation). We include NDVI change in the main text Figure 1 model 4 results as a potential alternative explanation for emigration resulting from livelihood losses. Over time **(B)**, NDVI responses to weather are evident (comparing to **Fig. S4**, panel B).

**
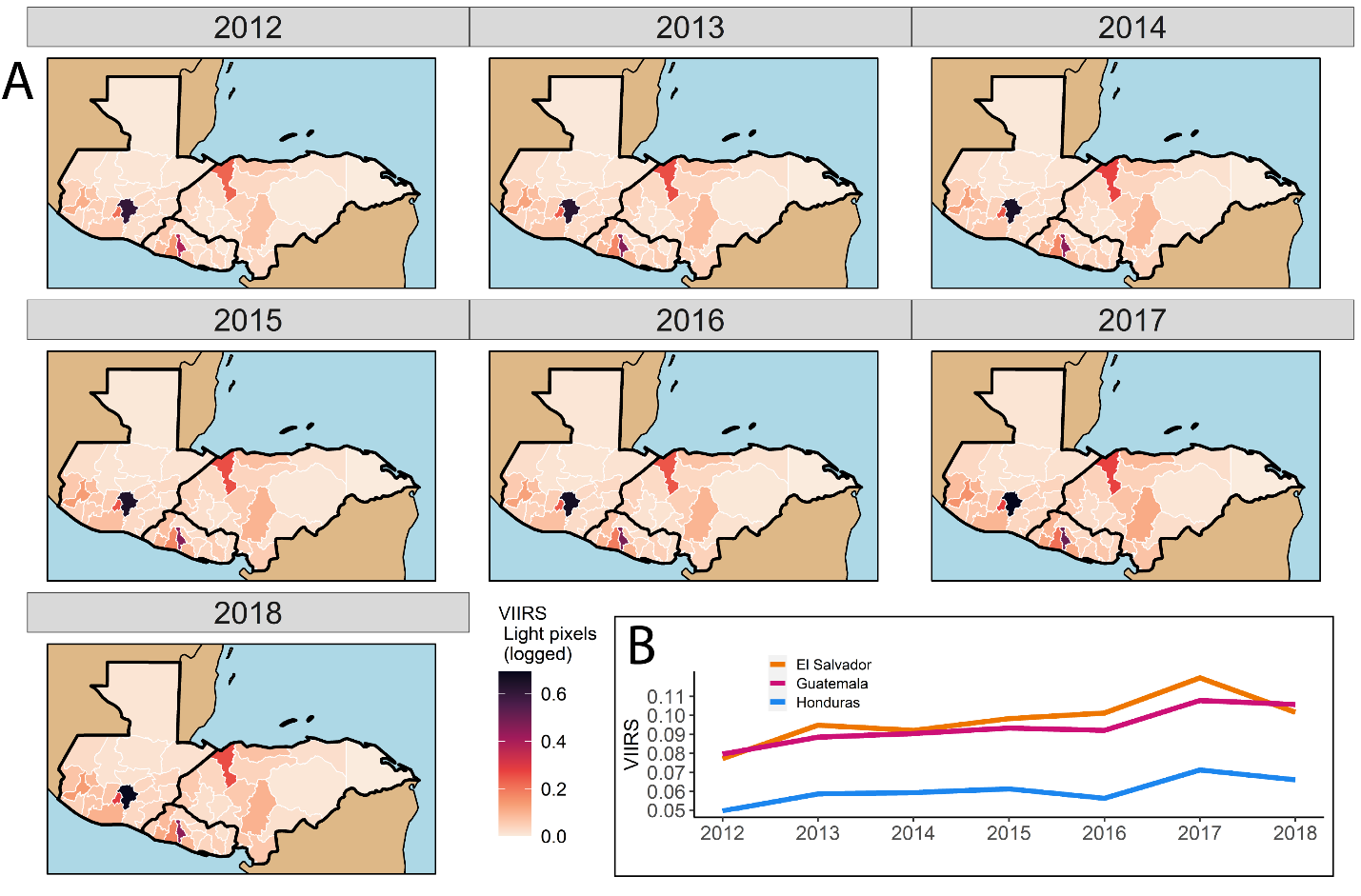
**

**Fig. S8.** By department and year **(A)**, VIIRS nighttime lights emissions. We include these data as a proxy for SES in the main text **Fig.1.** model 4 results. As expected, the regional trend since 2012 **(B)** is rising.


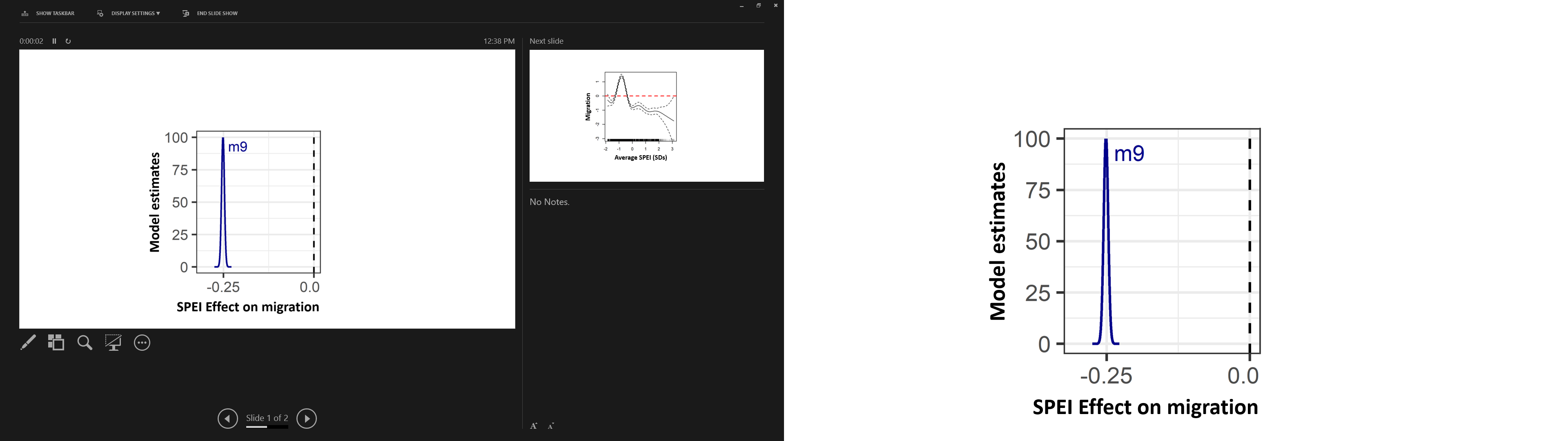


**Fig. S9.** A version of our main text **Fig. 1.** INLA model results with SPEI03 operationalized as a continuous variable rather than a dichotomous coding of dry conditions ≤ -1.0 SD. Model m9 just reflects this estimates addition to main text models m1-8.


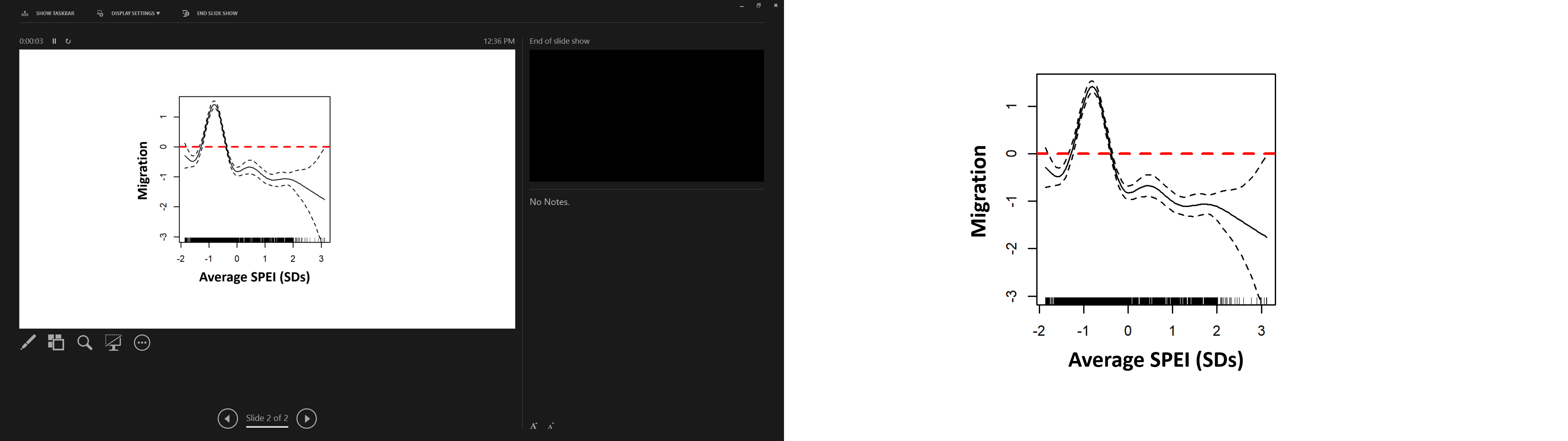


**Fig. S10.** Using the continuous variable operationalization of SPEI03, the effects of weather deviations from the historical average on migration.

| **Table S1.** Summary statistics for all independent and dependent variables in NTCA departments. | | | | | |
| --- | --- | --- | --- | --- | --- |
|  | **Mean** | **StdDev** | **Max** | **Min** | **Used in** |
| Emigration (Number of people) | 624.49 | 1045.65 | 10241.00 | 0.00 | m1-8 |
| Emigration rate (Per 100k) | 126.02 | 168.59 | 1005.28 | 0.00 | m9-12 |
| Emigration rate change | 32.79 | 140.70 | 795.86 | -574.74 | m7,m10,m12 |
| Homicide rate | 50.73 | 34.38 | 196.99 | 0.70 | m3,m4,m8 |
| Dry growing season SPEI03 | 0.13 | 0.34 | 1.00 | 0.00 | m1-4,m7-12 |
| Dry growing season SPEI12 | 0.19 | 0.40 | 1.00 | 0.00 | m6 |
| Very dry growing season SPEI03 | 0.02 | 0.14 | 1.00 | 0.00 | m5 |
| NDVI change (AVHRR) | -32.25 | 192.39 | 402.31 | -555.90 | m4-8 |
| Cropland area (ASAP) | 50.92 | 14.80 | 78.33 | 22.23 | m4-8 |
| Nighttime lights (VIIRS) | 0.49 | 0.88 | 5.89 | 0.00 | m4-8 |
| Population | 560503.06 | 536909.69 | 3489142.38 | 53920.14 | m4-8 |
| *Notes: Department N = 54; department-year spatial panel dataset N = 378.* | | | | |  |

| **Table S2.** Summary statistics for all independent and dependent variables in NTCA municipalities. | | | | | |
| --- | --- | --- | --- | --- | --- |
|  | **Mean** | **StdDev** | **Max** | **Min** | **Used in** |
| Emigration (Number of people) | 38.65 | 141.11 | 4163.00 | 0.00 | m8 |
| Homicide rate | 48.83 | 90.09 | 3073.29 | 0.00 | m8 |
| Dry growing season SPEI03 | 0.14 | 0.35 | 1.00 | 0.00 | m8 |
| NDVI change (AVHRR) | -36.21 | 252.93 | 1035.25 | -1572.95 | m8 |
| Cropland area (ASAP) | 47.95 | 20.17 | 98.06 | 4.75 | m8 |
| Nighttime lights (VIIRS) | 0.63 | 2.05 | 43.73 | 0.00 | m8 |
| Population | 34649.72 | 71749.29 | 1157509.20 | 637.00 | m8 |
| *Notes: Municipality N = 891; municipality-year spatial panel dataset N = 6237.* | | | | |  |

| **Table S3.** Point estimates and 95% credibility interval for all main text Figure 1 model 1 results. | | | |
| --- | --- | --- | --- |
|  | **Mean** | **95% Credibility interval** | |
| *Fixed effects* |  |  |  |
| (Intercept) | -1.163 | -1.462 | -0.865 |
| Dry growing season SPEI03 | 0.757 | 0.739 | 0.774 |
| *Random effect hyperparameters* |  |  |  |
| Precision for department (IID) | 0.834 | 0.560 | 1.189 |
| Precision for department (spatial) | 2138.063 | 237.058 | 7016.603 |
| Precision for year (temporal) | 258.764 | 71.296 | 586.315 |
| Precision for year (IID) | 0.485 | -0.113 | 0.892 |
| DIC | 32729.094 |  |  |
| *Notes: BYM model for department spatial random effects, AR1 model for yearly temporal correlation.* | | | |
|  |  |  |  |
| **Table S4.** Point estimates and 95% credibility interval for all main text Figure 1 model 2 results. | | | |
|  | **Mean** | **95% Credibility interval** | |
| *Fixed effects* |  |  |  |
| (Intercept) | 0.533 | 0.433 | 0.634 |
| Dry growing season SPEI03 | 0.944 | 0.926 | 0.963 |
| *Random effect hyperparameters* |  |  |  |
| Precision for department (IID) | 7.347 | 4.907 | 10.421 |
| Precision for department (spatial) | 1964.260 | 167.591 | 6828.943 |
| Precision for year (temporal) | 1601.692 | 452.660 | 3716.136 |
| Precision for year (IID) | 0.247 | -0.315 | 0.741 |
| DIC | 40325.574 |  |  |
| *Notes: BYM model for department spatial random effects, AR1 model for yearly temporal correlation.* | | | |
|  |  |  |  |
|  |  |  |  |
| **Table S5.** Point estimates and 95% credibility interval for all main text Figure 1 model 3 results. | | | |
|  | **Mean** | **95% Credibility interval** | |
| *Fixed effects* |  |  |  |
| (Intercept) | -1.549 | -1.820 | -1.278 |
| Dry growing season SPEI03 | 0.427 | 0.407 | 0.446 |
| Homicide rate | 0.009 | 0.008 | 0.009 |
| *Random effect hyperparameters* |  |  |  |
| Precision for department (IID) | 1.007 | 0.676 | 1.434 |
| Precision for department (spatial) | 1938.231 | 203.952 | 6353.359 |
| Precision for year (temporal) | 241.330 | 64.221 | 551.113 |
| Precision for year (IID) | 0.495 | -0.100 | 0.893 |
| DIC | 29231.617 |  |  |
| *Notes: BYM model for department spatial random effects, AR1 model for yearly temporal correlation.* | | | |
|  |  |  |  |
| **Table S6.** Point estimates and 95% credibility interval for all main text Figure 1 model 4 results. | | | |
|  | **Mean** | **95% Credibility interval** | |
| *Fixed effects* |  |  |  |
| (Intercept) | 2.142 | -1.096 | 5.375 |
| Dry growing season SPEI03 | 0.534 | 0.514 | 0.553 |
| Homicide rate | 0.006 | 0.006 | 0.007 |
| Nighttime lights (VIIRS) | 1.619 | 1.554 | 1.684 |
| NDVI change (AVHRR) | -0.001 | -0.001 | -0.001 |
| Cropland area (ASAP) | 0.018 | -0.030 | 0.064 |
| *Random effect hyperparameters* |  |  |  |
| Precision for department (IID) | 0.251 | 0.165 | 0.360 |
| Precision for department (spatial) | 2243.418 | 218.890 | 7907.177 |
| Precision for year (temporal) | 347.535 | 105.388 | 794.997 |
| Precision for year (IID) | 0.451 | -0.200 | 0.870 |
| DIC | 27248.013 |  |  |
| *Notes: BYM model for department spatial random effects, AR1 model for yearly temporal correlation.* | | | |
|  |  |  |  |
| **Table S7.** Point estimates and 95% credibility interval for all main text Figure 1 model 5 results. | | | |
|  | **Mean** | **95% Credibility interval** | |
| *Fixed effects* |  |  |  |
| (Intercept) | 1.975 | -0.850 | 4.797 |
| Very dry growing season SPEI03 | 0.112 | 0.087 | 0.137 |
| Homicide rate | 0.009 | 0.009 | 0.009 |
| Nighttime lights (VIIRS) | 1.320 | 1.258 | 1.381 |
| NDVI change (AVHRR) | -0.001 | -0.001 | -0.001 |
| Cropland area (ASAP) | 0.014 | -0.027 | 0.055 |
| *Random effect hyperparameters* |  |  |  |
| Precision for department (IID) | 0.331 | 0.217 | 0.474 |
| Precision for department (spatial) | 2645.805 | 318.325 | 9918.749 |
| Precision for year (temporal) | 295.038 | 84.661 | 692.321 |
| Precision for year (IID) | 0.488 | -0.184 | 0.898 |
| DIC | 28656.954 |  |  |
| *Notes: BYM model for department spatial random effects, AR1 model for yearly temporal correlation.* | | | |
|  |  |  |  |
| **Table S8.** Point estimates and 95% credibility interval for all main text Figure 1 model 6 results. | | | |
|  | **Mean** | **95% Credibility interval** | |
| *Fixed effects* |  |  |  |
| (Intercept) | 2.234 | -0.644 | 5.110 |
| Dry growing season SPEI12 | 0.086 | 0.070 | 0.102 |
| Homicide rate | 0.009 | 0.009 | 0.009 |
| Nighttime lights (VIIRS) | 1.338 | 1.276 | 1.400 |
| NDVI change (AVHRR) | -0.001 | -0.001 | -0.001 |
| Cropland area (ASAP) | 0.015 | -0.027 | 0.056 |
| *Random effect hyperparameters* |  |  |  |
| Precision for department (IID) | 0.318 | 0.209 | 0.457 |
| Precision for department (spatial) | 2063.231 | 270.822 | 6899.781 |
| Precision for year (temporal) | 291.822 | 97.167 | 634.065 |
| Precision for year (IID) | 0.505 | -0.083 | 0.884 |
| DIC | 28486.367 |  |  |
| *Notes: BYM model for department spatial random effects, AR1 model for yearly temporal correlation.* | | | |
|  |  |  |  |
| **Table S9.** Point estimates and 95% credibility interval for all main text Figure 1 model 7 results. | | | |
|  | **Mean** | **95% Credibility interval** | |
| *Fixed effects* |  |  |  |
| (Intercept) | 0.384 | 0.280 | 0.488 |
| Dry growing season SPEI03 | 0.173 | 0.129 | 0.216 |
| Homicide rate | 0.000 | 0.000 | 0.000 |
| Nighttime lights (VIIRS) | -0.005 | -0.017 | 0.007 |
| NDVI change (AVHRR) | 0.000 | 0.000 | 0.000 |
| Cropland area (ASAP) | 0.000 | -0.001 | 0.001 |
| *Random effect hyperparameters* |  |  |  |
| Precision for the Gaussian observations | 130.766 | 111.050 | 152.559 |
| Precision for department (IID) | 6475.083 | 2412.903 | 14228.435 |
| Precision for department (spatial) | 2596.206 | 369.148 | 8569.959 |
| Precision for year (temporal) | 56209.662 | 11685.446 | 170088.073 |
| Precision for year (IID) | 0.651 | -0.268 | 0.973 |
| DIC | -634.981 |  |  |
| *Notes: BYM model for department spatial random effects, AR1 model for yearly temporal correlation.* | | | |
|  |  |  |  |
| **Table S10.** Point estimates and 95% credibility interval for all main text Figure 1 model 8 results. | | | |
|  | **Mean** | **95% Credibility interval** | |
| *Fixed effects* |  |  |  |
| (Intercept) | 0.612 | 0.219 | 1.005 |
| Dry growing season SPEI03 | 0.367 | 0.354 | 0.381 |
| Homicide rate | 0.002 | 0.002 | 0.002 |
| Nighttime lights (VIIRS) | 0.061 | 0.052 | 0.070 |
| NDVI change (AVHRR) | 0.000 | 0.000 | 0.000 |
| Cropland area (ASAP) | 0.005 | 0.001 | 0.009 |
| *Random effect hyperparameters* |  |  |  |
| Precision for municipality (IID) | 1.660 | 1.096 | 2.475 |
| Precision for municipality (spatial) | 0.031 | 0.022 | 0.042 |
| Precision for year (temporal) | 34394.975 | 8993.192 | 86507.245 |
| Precision for year (IID) | 0.681 | 0.043 | 0.958 |
| DIC | 96017.902 |  |  |
| *Notes: BYM model for municipality spatial random effects, AR1 model for yearly temporal correlation.* | | | |
|  |  |  |  |
| **Table S11.** Point estimates and 95% credibility interval for a model testing an alternative operationalization of the homicide rate variable. | | | |
|  | **Mean** | **95% Credibility interval** | |
| *Fixed effects* |  |  |  |
| (Intercept) | 1.849 | -1.960 | 5.653 |
| Dry growing season SPEI03 | 0.739 | 0.721 | 0.757 |
| Higher than average homicide rate | 0.209 | 0.194 | 0.225 |
| Nighttime lights (VIIRS) | 2.061 | 1.999 | 2.124 |
| NDVI change (AVHRR) | -0.001 | -0.001 | -0.001 |
| Cropland area (ASAP) | 0.022 | -0.034 | 0.077 |
| *Random effect hyperparameters* |  |  |  |
| Precision for department (IID) | 0.181 | 0.119 | 0.260 |
| Precision for department (spatial) | 1936.168 | 152.057 | 7165.001 |
| Precision for year (temporal) | 356.050 | 104.842 | 830.260 |
| Precision for year (IID) | 0.454 | -0.216 | 0.871 |
| DIC | 27919.128 |  |  |
| *Notes: BYM model for department spatial random effects, AR1 model for yearly temporal correlation.* | | | |
